# Exosomes derived from mycobacterium tuberculosis-infected MSCs induce a pro-inflammatory response of macrophages

**DOI:** 10.18632/aging.202854

**Published:** 2021-04-19

**Authors:** Min Liu, Zaiguo Wang, Shaolei Ren, Hongli Zhao

**Affiliations:** 1 Jinan People’ s Hospital Affiliated to Shandong First Medical University, Laiwu, Shandong Province, China; 2 Department of Critical Care Medicine, Penglai Hospital of Traditional Chinese Medicine, Penglai, Shandong Province, China; 3 Penglai Hospital of Traditional Chinese Medicine, Penglai, Shandong Province, China; 4 Department of Senile Diseases, Dongying City Shengli Hospital, Dongying, Shandong Province, China

**Keywords:** mesenchymal stem cell, exosome, mycobacterium tuberculosis, pro-inflammatory response, toll-like receptor

## Abstract

Tuberculosis (TB) is a common infectious disease caused by Mycobacterium tuberculosis (M.tb), and macrophages serve as the primary natural host of M.tb. Mesenchymal stem cells (MSCs)-derived exosomes play an essential role in inflammatory responses. This study aimed to determine the role of exosomes derived from M.tb-infected MSCs (Exo-MSCs-M.tb) on macrophages *in vitro* and *in vivo* and the underlying mechanisms. Here, we demonstrated that M.tb infection promoted the production of Exo-MSCs-M.tb, but did not influence MSCs proliferation. Exo-MSCs-M.tb were taken up by macrophages and then induced the pro-inflammatory response of macrophages through elevating the production of TNF-α, RANTES, and iNOS. Also, pro-inflammatory response induced by Exo-MSCs-M.tb displayed a time-dependent pattern in macrophages, in which the highest level of inflammatory response was observed at 72 hours post-infection of MSCs. In addition, the effect of Exo-MSCs-M.tb was mediated through TLR2/4 and MyD88 signaling pathways. Furthermore, Exo-MSCs-M.tb could induce the pro-inflammatory response in mice *in vivo*, and exosomes isolated from Exo-MSCs-M.tb-treated mice could also promote the pro-inflammatory response. Taken together, these results indicate that Exo-MSCs-M.tb induced the pro-inflammatory response of macrophages through TLRs signaling. This study provides new insight into the potential of MSCs-derived exosomes for the treatment of TB.

## INTRODUCTION

Tuberculosis (TB) is a common infectious disease caused by Mycobacterium tuberculosis (M.tb), an obligate intracellular organism [1]. TB is a highly heterogenetic and variable disease, leading to a wide range of clinical manifestations in humans [2]. In 2017, it was estimated that 1.6 million patients who died from TB, including 300,000 cases are associated with human immunodeficiency virus (HIV) [3]. It has been reported that a large proportion of patients with TB are resistant to rifampicin, which is the most effective first-line drug [4]. To date, the widespread of drug-resistant TB, along with the HIV epidemic, make it more challenging for TB treatment and management [5]. Therefore, developing new diagnostic approaches and effective drugs, or combinational therapies, are urgently needed for TB patients [6].

The mechanism underlying the drug resistance of M.tb remains to be thoroughly investigated. Antibiotic-based treatments are aimed to inhibit the replication of M.tb [7]. Macrophages serve as a natural host of M.tb, in which M.tb can replicate and survive through various host-evasion strategies, including translocation to the cytosol [8], deacidification of lysosomal components [9], and suppression of phagolysosome fusion [10]. On the other hand, drug-resistant bacteria can make use of granulomatous structures as a shelter to survive, which are rarely accessible to drugs [11]. Also, within the granulomatous structures, mesenchymal stem cells (MSCs), multi-potential progenitor cells, are effectively recruited by M.tb at the infectious site and play an immunosuppressive role to inhibit the T cell response through generating the nitric oxide (NO), tumor necrosis factor-α (TNF-α), and C-C Motif Ligand-5 (RANTES) [12, 13]. Growing evidence suggests that MSCs are a reservoir of M.tb infection, playing an antibiotic-protective role for M.tb [14]. However, the detailed mechanism underlying the immunosuppressive effect of MSCs remain unknown.

Exosomes are membrane-enclosed vesicles with diameters of 30-150 nm and can be released by almost all cell types [15]. Exosomes play a critical role in intercellular communication through transporting various molecular components, such as lipids, enzymes, microRNAs, and proteins, eventually regulating cellular behaviors of recipient cells [16]. MSCs-derived exosomes perform a wide variety of jobs in the inflammatory response, neurogenesis, cardiovascular diseases, and tumor growth [17]. Zhang et al. reported that MSCs-derived exosomes attenuate temporomandibular joint osteoarthritis through suppressing inflammatory response and restoring joint homeostasis [18]. Teng et al. demonstrated that exosomes derived from MSCs improve the heart function of infarcted myocardium through stimulating neovascularization and restraining inflammation response [19]. Also, Cosenza et al. reported that MSCs-derived exosomes play an anti-inflammatory role on T and B lymphocytes in inflammatory arthritis [20].

Given the importance of MSCs-derived exosomes in the inflammatory response, we aimed to investigate the effect of exosomes derived from M.tb-infected MSCs (Exo-MSCs-M.tb) on macrophages and the related mechanism. In this study, we demonstrated that Exo-MSCs-M.tb promoted the pro-inflammatory response of macrophages *in vitro*. Also, toll-like receptor 2/4 (TLR2/4), pattern recognition receptors (PRRs), were associated with the effect of exosomes on macrophages. Moreover, Exo-MSCs-M.tb could activate the inflammatory response in mice *in vivo*.

## MATERIALS AND METHODS

### Ethics statement

In the study, human studies were approved ethically by the Ethics Committee of Penglai Hospital of Traditional Chinese Medicine, and the experiments were carried out in accordance with the Animal Declaration of Helsinki Principles. The participants provided their written informed consent to participate in this study. Animal experiments were performed in accordance with the guidelines approved by the Institutional Care and Use Committee of Penglai Hospital of Traditional Chinese Medicine.

### MSCs isolation and differentiation

Bone marrow was collected from 5 healthy human donors (2 males and 3 females, aged 23-31 years). MSCs were isolated from bone marrow samples, as previously described [21]. Briefly, bone marrow cells were flushed and centrifuged in a 1.073 g/ml percoll density gradient (Pharmacia, USA). Then, the enriched cells were harvested from the interphase, and resuspended in culture medium, transferred into culture flasks with low glucose containing Dulbecco's Modified Eagle's Medium (LG-DMEM; Gibco, USA) supplemented with 10% fetal bovine serum (FBS; Gibco, USA) in humid air with 5% CO_2_ at 37° C. After reaching confluence, cells were collected and cryopreserved as primary MSCs. For subsequent experiments, MSCs at the third passage were used throughout the study, and cells were seeded at the required cell density, 24 hours before the experiments. To identify MSCs, cells were stained with positive MSC surface markers CD29, CD44, CD73, CD90, CD271, and CD105, and negative markers CD31, CD45, and HLA-DR (Abcam, China). Flow cytometry analysis was carried out on Cytomics FC500 MPL Flow Cytometer (Beckman Coulter, USA). Meanwhile, the differentiation of MSCs to adipocytes and chondrocytes was determined by using the adipogenesis differentiation kit (Gibco, USA) and StemPro™ Chondrogenesis Differentiation Kit (Gibco, USA) according to the manufacturer's instruction. Afterward, Oil Red O and Alcian Blue staining were carried out to determine adipocytes and chondrocytes, respectively [22].

### Bacterial culture

The M.tb H37Rv strain was obtained from American Type Culture Collection and grown in a Middlebrook 7H9 medium (BD Biosciences, USA) supplemented with 10% Ovalbumin, Dextrose and Catalase (OADC; Difco^TM^, USA), 0.2% glycerol and 0.05% Tween-80. Bacterial cultures were cryopreserved in 20% glycerol and preserved at -80° C for subsequent experiments.

### M.tb infection and confocal microscopy

MSCs were infected with M.tb at 1:50 multiplicity of infection (MOI) for 6 hours [23, 24]. Post-infection, MSCs were treated with 100 μg/ml Gentamicin (Abcam, China) for 2 hours, and then were washed and incubated in LG-DMEM (Gibco, USA) in humid air with 5% CO_2_ at 37° C. To visualize M.tb infection, MSCs were seeded on glass coverslips and infected with M.tb-GFP at 1:50 MOI. After 6 hours of infection, MSCs were washed three times with media supplemented with 10% FBS and treated with 100 μg/ml 100 μg/ml Gentamicin (Abcam, China) for 2 hours. Next, M.tb-infected MSCs were washed twice with fresh media and placed in humid air with 5% CO_2_ at 37° C. After 72 hours of infection, MSCs were washed three times with PBS solution buffer and fixed in 4% paraformaldehyde in PBS solution for 20 min. After fixation, MSCs were washed three times with PBS solution buffer and placed at 4° C. Afterward, MSCs were stained with DAPI (Invitrogen, USA) according to the manufacturer's instruction and were observed under a Leica confocal 118 SP5 confocal microscope (Leica, Germany).

### Exosome isolation and identification

After 72 hours of infection, M.tb-infected MSCs-conditioned medium was collected to isolate exosomes. Exo-MSCs-M.tb were isolated using ExoQuick-TC™ Exosome Precipitation Solution (System Biosciences, USA) according to the manufacturer's instruction. Isolated exosomes were dissolved in PBS solution buffer and stored at -80° C. Protein in exosomes was isolated using RIPA lysis buffer (Thermo Fisher Scientific, USA) and used to quantify exosomal protein using BCA Protein Assay Kit (Thermo Fisher Scientific, USA). The concentration and size of exosomes were measured through transmission electron microscopy (TEM) and nanoparticle tracking analysis (NTA) with a Hitachi H-600 TEM microscope (Hitachi, USA), as previously described [25, 26]. Exosomal surface markers CD63, CD81, Hsp70, Hsp90, and TSG101, were identified using flow cytometric analysis on Cytomics FC500 MPL Flow Cytometer (Beckman Coulter, USA), as previously described [27, 28]. The MSCs-conditioned medium was harvested at indicated times for subsequent experiments. The concentration of exosomal protein was detected using BCA Protein Assay Kit (Abcam, China) according to the manufacturer's instructions.

### Macrophages culture

Human macrophage cell line THP-1 was obtained from MilliporeSigma (MilliporeSigma, USA). Macrophages were cultured in RPMI 1640-GlutaMAX™ medium supplemented with 10% FBS, 10 mM pyruvate carbonate, and 10 mM Na^+^ HEPES in humid air with 5% CO_2_ at 37° C. Macrophages were subjected to 200 nM phorbol 12-myristate 13-acetate (PMA; Sigma-Aldrich, China) to differentiate into macrophages 24 hours prior to exosome treatment. Macrophages were cultured on non-tissue culture plates for 3 days and removed by PBS solution buffer (10 mM EDTA), and then seeded in tissue-culture plates (1×10^5^) per well for 24 hours prior to exosome or apoptotic vesicle treatment. 20 μg exosomes or apoptotic vesicles were used to treat macrophages for 24 hours and then lysed using lysis buffer (Sigma-Aldrich, China). For caspase-3 inhibition, 50 μM caspase-3 inhibitor DEVD-CHO (BD Pharmingen™, USA) was added to macrophages for 1 hour prior to exosome treatment.

### BODIPY 493/503 staining assay

BODIPY 493/503 staining was performed using BODIPY™ 493/503 fluorescent probe (Thermo Fisher Scientific, USA) as previously described [29]. BODIPY 493/503 was diluted in 10% (v/v) dimethyl sulfoxide to a concentration of 1 mg/mL. After 72 hours of infection, M.tb-infected MSCs were fixed with 4% paraformaldehyde for 20 min and then incubated with BODIPY 493/503 at 37° C for 30 min. Afterward, cells were with PBS solution three times. The images were taken using Leica confocal 118 SP5 confocal microscope (Leica, Germany).

### 5-Ethynyl-2′-deoxyuridine (EdU) assay

After 72 hours of infection, cell DNA synthesis and cell proliferation were investigated in M.tb-infected MSCs using EdU assay kit (RiboBio, China) according to the manufacturers' instructions. Images were taken using Leica confocal 118 SP5 confocal microscope (Leica, Germany).

### ELISA

ELISA assay was performed to measure the levels of TNF-α, RANTES, and IL-12p40 in macrophage-conditioned medium or the homogenates of lung tissues (Invitrogen, USA) 24 hours after exosome treatment. Absorbance was detected at 450 nm using the Spectra Max 190 ELISA reader (Molecular Devices, USA). Five replicates were included in each sample, and the concentrations were quantified against the standard curve.

### Western blotting

Total proteins of cells or exosomes were isolated using RIPA peptide lysis buffer (Beyotime Biotechnology, China). 25 μg protein content was loaded in 10% Sodium Dodecyl Sulfate–Polyacrylamide Gel Electrophoresis (SDS-PAGE) gels, electrophoresed, and then transferred to polyvinylidene difluoride membranes (MilliporeSigma, USA). The membranes were incubated with primary antibodies against inducible nitric oxide synthase (iNOS), 19-kDa lipoprotein, lipoarabinomannan (LAM), Hsp70, major histocompatibility complex class II (MHC II), phospho-IκBα (p-IκBα), phospho-p38 (p-p38), total p38 (p38), toll-like receptor 2 (TLR2), toll-like receptor 4 (TLR4), Myeloid differentiation factor 88 (MyD88), and GAPDH (Abcam, China) overnight at 4° C. After washing with TBST 3 times, the membranes were incubated with corresponding secondary antibodies for 1 hour. Protein bands were imaged and analyzed using the Chemiluminescent Substrate System (Thermo Fisher Scientific, USA).

### Immunofluorescence staining

Exosomes were labeled with the lipophilic dye DiO (Sigma-Aldrich, China) according to the manufacturer's instructions. The cytoskeleton of macrophages was labeled with TRITC Phalloidin according to the manufacturer's instructions (Sigma-Aldrich, China). Macrophages (1 × 10^5^) were seeded on coverslips and cocultured with labeled exosomes for 24 hours. Then, macrophages treated with exosomes were fixed with fixed in 4% paraformaldehyde in PBS solution, permeabilized with 0.1% Triton X-100, and blocked with 3% bovine serum albumin (BSA) (Thermo Fisher Scientific, China). Afterward, the coverslips were incubated with the primary antibodies overnight at 4° C and the secondary antibodies for 30 min in the dark. Then, the nuclei were stained with 4′,6-diamidino-2-phenylindole (DAPI; Sigma-Aldrich, China). The coverslips were imaged using were observed under a Leica confocal 118 SP5 confocal microscope (Leica, Germany).

### Apoptotic vesicles isolation

Apoptotic vesicles were isolated using serial centrifugation, as previously described [30]. Briefly, apoptosis was induced by the deprivation of fetal calf serum [31]. 72 hours after treatment, cell-conditioned medium was collected and centrifuged at 800g (15 min), 1800g (15 min), and 25000g (20 min). After removing the supernatant, the remaining was spun at the ultracentrifugation method (100000g) for 1h to pellet the apoptotic vesicles.

### Annexin V staining

To evaluate the apoptosis, macrophages treated with indicated treatments or underwent serum starvation were stained with annexin V using Annexin V-FITC Apoptosis Detection Kit (MilliporeSigma, USA) according to the manufacturer's instructions. Macrophages were imaged using were observed under a Leica confocal 118 SP5 confocal microscope (Leica, Germany).

### *In vivo* experiments

C57BL/6, TLR2^−/−^ (C57BL/6), TLR4^−/−^ (C57BL/10), and MyD88^−/−^ mice (male, 6-8 weeks old) were obtained from Nanjing Biomedical Research Institute of Nanjing University (Nanjing, China) and bred under pathogen-free environment. Mouse macrophages were isolated from TLR2^−/−^, TLR4^−/−^, and MyD88^−/−^ mice, as previously described [32]. C57BL/6 (n=10 each group) were injected intranasally with 50 μL PBS (control), 20 μg exosomes isolated from MSCs (Exo-MSCs) or M.tb-infected MSCs (Exo-MSCs-M.tb) in 50 μL PBS. After 3 days of injection, mice were sacrificed, and the lungs were lavaged with sterile PBS buffer solution four times. Then, the lungs were collected and homogenized in 5 mL of 1% NP-40 solution (1%), and 1 mL of lung homogenate was used for the levels of IL-12P40 and TNF-α using ELISA assay. The number of neutrophils per 100 bronchoalveolar lavage fluid (BALF) cells was defined. Also, exosomes were isolated from BALF of mice injected with Exo-MSCs-M.tb, and were identified through TEM, NTA, and flow cytometric analysis.

### Statistical analysis

Data are presented as means ± SEM. Statistical analysis was carried out using SPSS v.19.0 software (SPSS, USA). Differences between groups were analyzed with one-way ANOVA or Student's t-test. Differences were considered to be significant at P < 0.05.

## RESULTS

### MSCs identification

**Figure 1:**
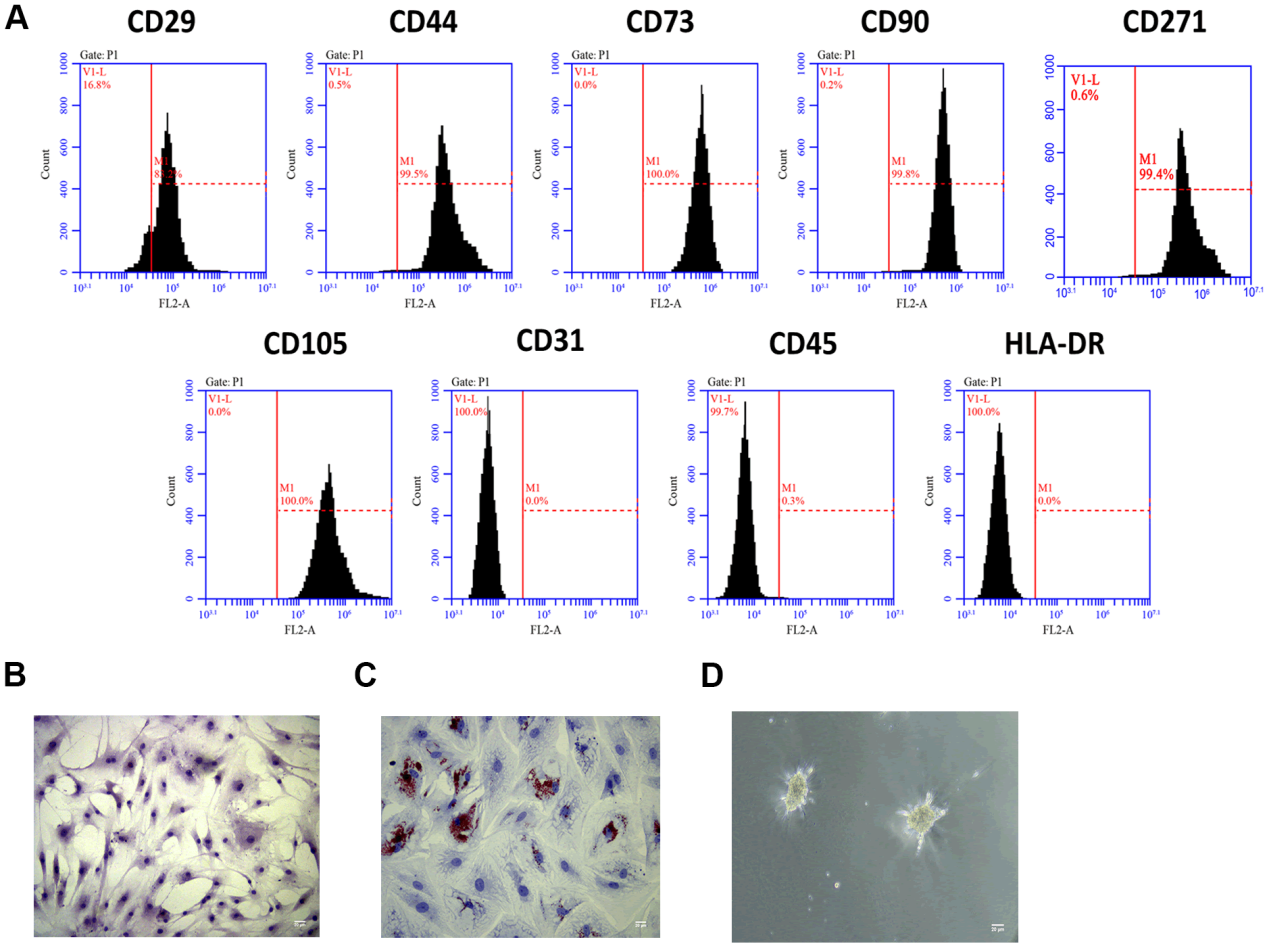
**MSCs identification.** (**A**) Detection of stem cell markers by flow cytometer. MSCs positively expressed CD29, CD44, CD73, CD90, CD271 and CD105, while negatively expressed CD31, CD45, and HLA-DR. (**B**) Morphology of MSCs under an inverted microscope. Scale bar: 20 μm. (**C**) Adipogenic differentiation of MSCs, as detected by oil red O staining. Scale bar: 20 μm. (**D**) Chondrogenesis differentiation of MSCs, as detected by Alcian Blue staining. Scale bar: 20 μm.

To investigate the role of Exo-MSCs-M.tb in the pro-inflammatory response of macrophages, we isolated MSCs from the human bone marrow. Through flow cytometry analysis, MSCs surface markers CD29, CD44, CD73, CD90, CD271, and CD105 were positively expressed, while the expressions of CD31, CD45, and HLA-DR were rarely expressed in MSCs (Figure 1A). Also, the morphology of MSCs was determined by an inverted microscope (Figure 1B). Positive staining of Oil Red (31.2%) was found after adipogenic induction of MSCs for 14 days (Figure 1C), and positive staining of Alcian Blue was observed after chondrogenic induction of MSCs for 28 days (Figure 1D). Collective, we successfully identified MSCs, and they could be used for subsequent experiments.

### MSCs were infected with M.tb

**Figure 2:**
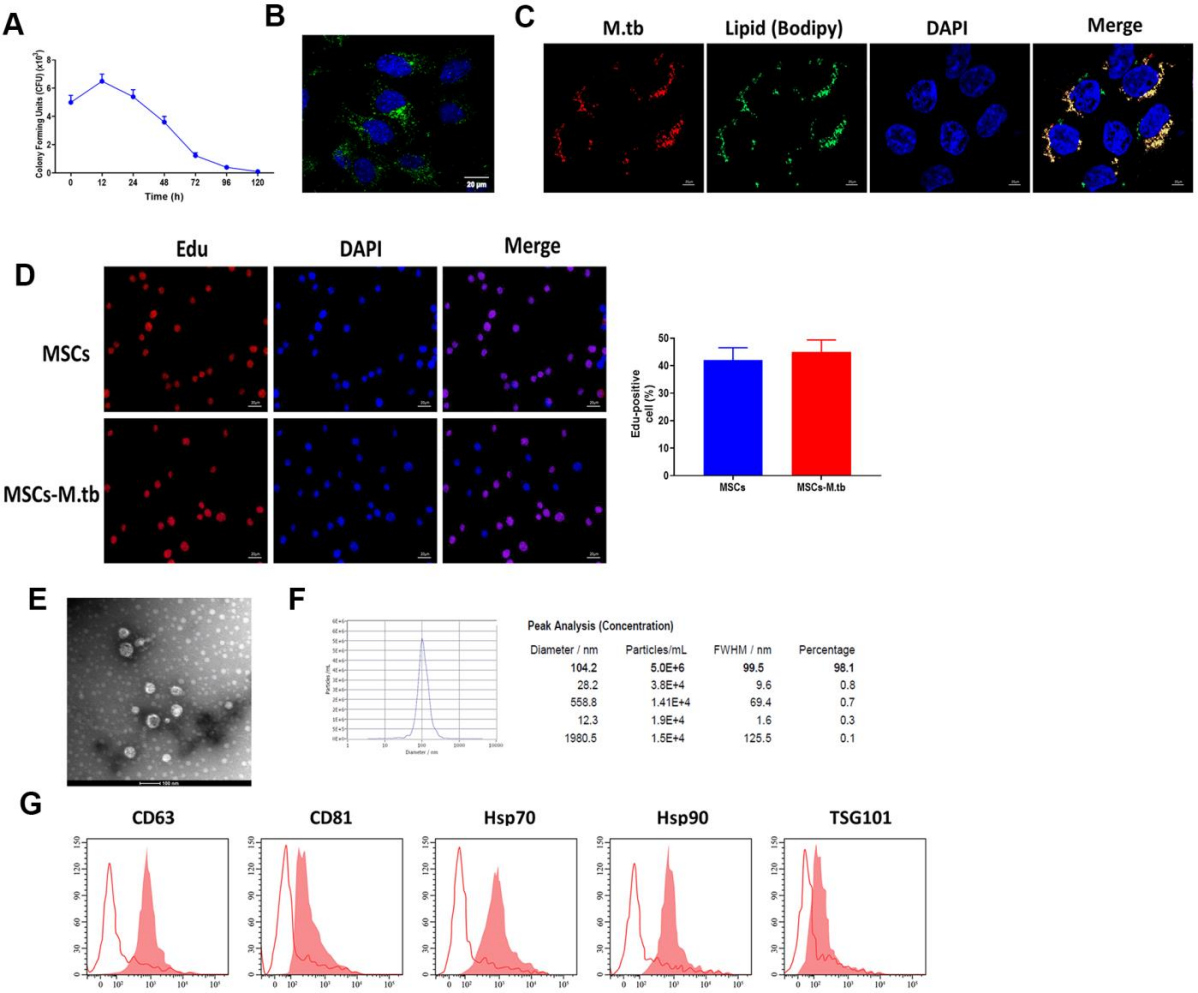
**MSCs were infected with M.tb.** (**A**) Colony forming units (CFU) of M.tb in MSCs. (**B**) MSCs were infected with M.tb for 72 hours, as imaged by confocal microscopy. Scale bars: 20 μm. Green color represents M.tb GFP. Blue color represents nuclei. (**C**) Bodipy 493/503 assay in MSCs infected with M.tb. Scale bars: 20 μm. Red color represents M.tb GFP. Green color represents neutral lipids. Blue color represents nuclei. (**D**) Edu assay in MSCs infected without or with M.tb. Scale bars: 20 μm. (**E**) Morphology of exosomes derived from M.tb infected MSCs, as imaged by TEM. Scale bars: 100 μm. (**F**) Concentration and diameter distribution of Exo-MSCs-M.tb, as detected by NTA. (**G**) Detection of exosomal markers CD63, CD81, Hsp70, Hsp90, and TSG101, as detected by flow cytometer.

Given the reference [23, 24], we attempted to obtain a saturation of infection in MSCs and the results revealed that 6 hours at 1:50 multiplicity of infection (MOI) attained saturation of infection of M.tb (Figure 2A). Under these conditions, M.tb was taken up by MSCs, as imaged by confocal microscopy (Figure 2B). By using BODIPY 493/503 staining assay, we found that M.tb colocalized with neutral lipids in MSCs (Figure 2C). Also, Edu assay revealed that M.tb infection did not influence the proliferation of MSC (Figure 2D). After 72-hour infection, the conditioned medium by M.tb-infected MSCs was used to isolate exosomes (Exo-MSCs-M.tb). By performing TEM and NTA assays, we found that M.tb-infected MSCs secreted spherical shape vesicles with a diameter of 104.2 nm (98.1%) (Figure 2E, 2F). Furthermore, flow cytometric analysis demonstrated that the vesicles were positive for exosomal surface markers CD63, CD81, Hsp70, Hsp90, and TSG101 (Figure 2G). Collectively, these results suggest that we successfully established M.tb-infected MSCs model.

### Exo-MSCs-M.tb were internalized by macrophages

**Figure 3:**
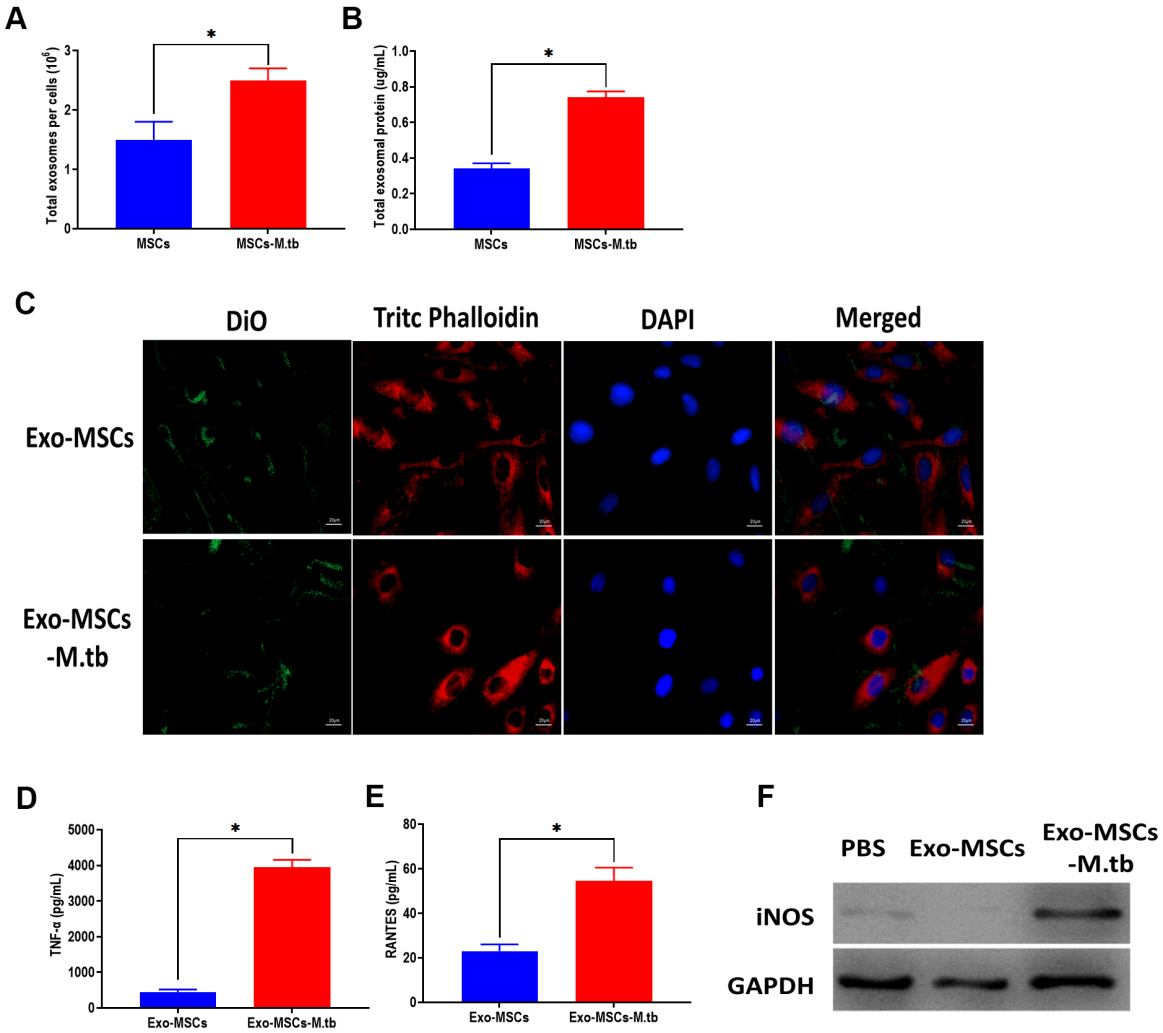
**Exo-MSCs-M.tb were internalized by macrophages.** (**A**) The number of exosomes released from M.tb-infected MSCs or PBS-treated control MSCs. (**B**) Exosomal protein level in M.tb-infected MSCs compared with those in PBS-treated control MSCs. (**C**) Exo-MSCs-M.tb (20μg) were internalized by macrophages, as imaged by confocal microscopy. Exosomes were stained by DiO (Green). Cytoskeleton was stained by TRITC Phalloidin (red). Nuclei were stained by DAPI (blue). Scale bar: 20 μm. (**D**) TNF-α levels in macrophages treated with Exo-MSCs-M.tb (20μg) or Exo-MSCs (20μg), as detected by ELISA assay 24 hours after exosome treatment. (**E**) RANTES levels in macrophages treated with Exo-MSCs-M.tb (20μg) or Exo-MSCs (20μg), as detected by ELISA assay 24 hours after exosome treatment. (**F**) iNOS levels in macrophages treated with with Exo-MSCs-M.tb (20μg) or Exo-MSCs (20μg), as detected by western blotting assay 24 hours after exosome treatment. *p < 0.05.

In this study, we measured the number of exosomes and exosomal protein concentrations in conditioned medium. The results showed that M.tb infection significantly increased exosome release in MSCs, compared with uninfected MSCs (Figure 3A, 3B). Next, we aimed to determine the role of Exo-MSCs-M.tb in macrophages. As displayed in Figure 3C, Exo-MSCs and Exo-MSCs-M.tb labeled with green DiO were internalized by macrophages. Also, we found that Exo-MSCs-M.tb increased the levels of tumor necrosis factor-α (TNF-α), RANTES, and iNOS production of macrophages, compared with Exo-MSCs (Figure 3D–3F). These results indicate that Exo-MSCs-M.tb might be associated with the pro-inflammatory activity of macrophages.

### Exo-MSCs-M.tb induced the pro-inflammatory response of macrophages

**Figure 4:**
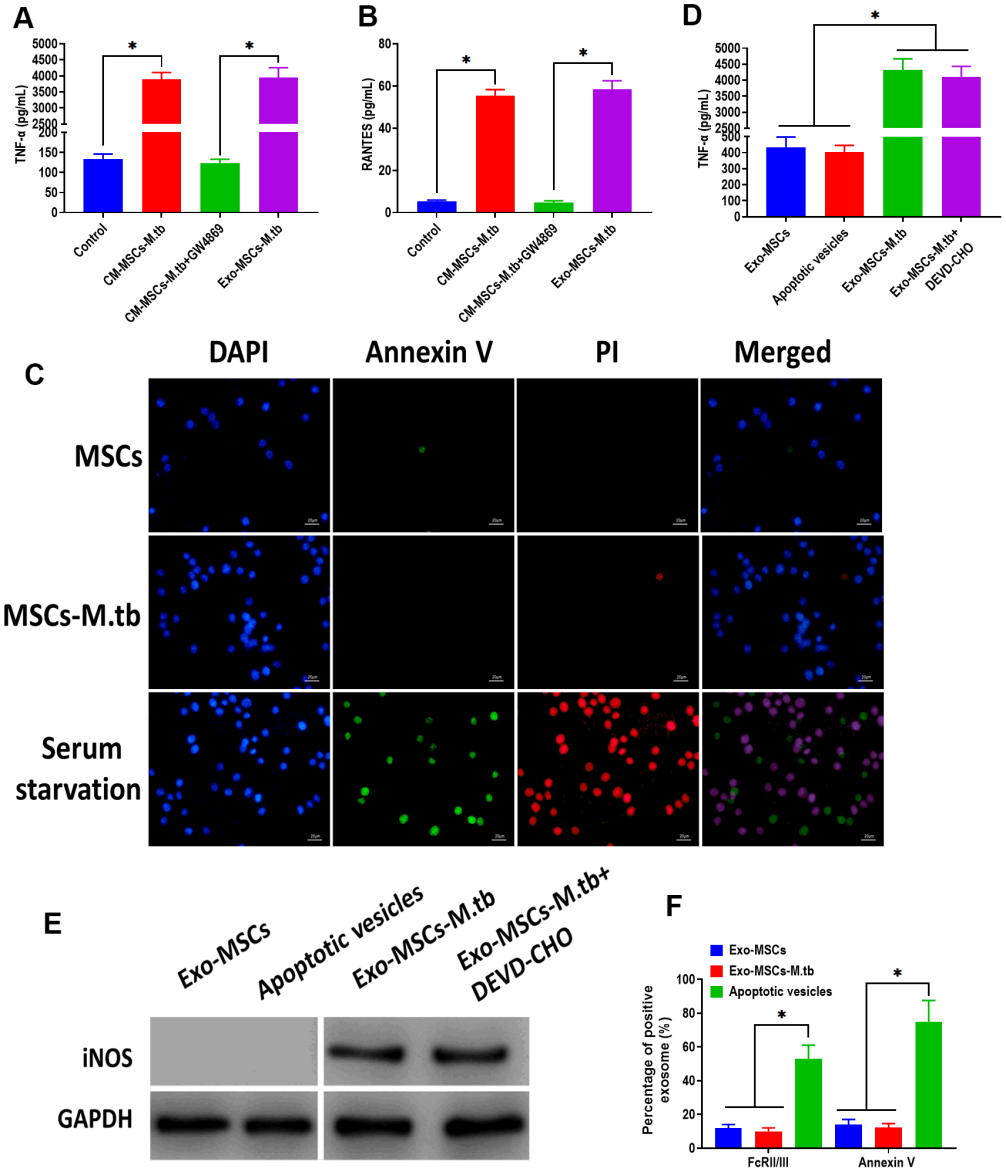
**Exo-MSCs-M.tb induced pro-inflammatory response of macrophages.** (**A**) TNF-α levels in macrophages treated with M.tb-infected MSCs-conditioned medium (CM-MSCs-M.tb), M.tb-infected MSCs-CM plus GW4869, and Exo-MSCs-M.tb (20μg). (**B**) RANTES-α levels in macrophages treated with M.tb-infected MSCs-CM, M.tb-infected MSCs-CM plus GW4869, and Exo-MSCs-M.tb (20μg). (**C**) Apoptotic level of macrophages treated with PBS (control), Exo-MSCs-M.tb (20μg), and serum-deprivation treatment. Macrophages were stained with DAPI (blue), annexin V (green), and PI (red). Scale bar: 20 μm. (**D**) TNF-α levels in macrophages treated with apoptotic vesicles, Exo-MSCs-M.tb (20μg), and Exo-MSCs-M.tb plus DEVD-CHO (caspase-3 inhibitor), as detected by ELISA assay 24 hours after exosome treatment. (**E**) iNOS levels in macrophages treated with apoptotic vesicles, Exo-MSCs-M.tb (20μg), and Exo-MSCs-M.tb plus DEVD-CHO (caspase-3 inhibitor), as detected by western blotting assay. (**F**) Annexin V and FcRII/III levels in exosomes derived from MSCs or M.tb-infected MSCs, and apoptotic vesicles, as detected by flow cytometer. *p < 0.05.

To further investigate that the pro-inflammatory response was induced by Exo-MSCs-M.tb, not mycobacterial components, GW4869, a pharmacological agent for blocking exosome generation [33], was added to the medium of M.tb-infected MSCs. We found that Exo-MSCs-M.tb and conditioned medium of M.tb-infected MSCs (CM-MSCs-M.tb) significantly increased the level of TNF-α and RANTES of macrophages, which was reversed by the addition of GW4869 (Figure 4A, 4B). These results suggest that Exo-MSCs-M.tb might be a major component to induce the pro-inflammatory response. To determine whether the M.tb infection caused apoptosis, MSCs and M.tb-infected MSCs were stained with annexin A, an indicator of surface-exposed phosphatidylserine, and with propidium iodide (PI), an indicator of late apoptosis [34]. As shown in Figure 4C, the negative staining of annexin A and PI were observed in MSCs and M.tb-infected MSCs, whereas the positive staining of both was found in MSCs underwent serum starvation-induced apoptosis. Moreover, to eliminate the possibility that apoptotic vesicles might lead to the pro-inflammatory response of macrophages, we measured the level of TNF-α and RANTES of macrophages treated with apoptotic vesicles isolated from serum starvation-treated MSCs. The results demonstrated that apoptotic vesicles could not induce the pro-inflammatory response of macrophages, while Exo-MSCs-M.tb isolated from MSCs treated with or without DEVD-CHO, caspase-3 inhibitor [35], displayed a similar role in activating the pro-inflammatory response of macrophages (Figure 4D, 4E). Furthermore, staining of FcRII/III and annexin V displayed a higher level in apoptotic vesicles isolated from serum starvation-treated MSCs than both Exo- MSCs-M.tb and Exo-MSCs (Figure 4F). Together, these results indicate that Exo-MSCs-M.tb was the primary inducer for the pro-inflammatory response of macrophages.

### Pro-inflammatory response induced by Exo-MSCs-M.tb were time-dependent in macrophages

**Figure 5:**
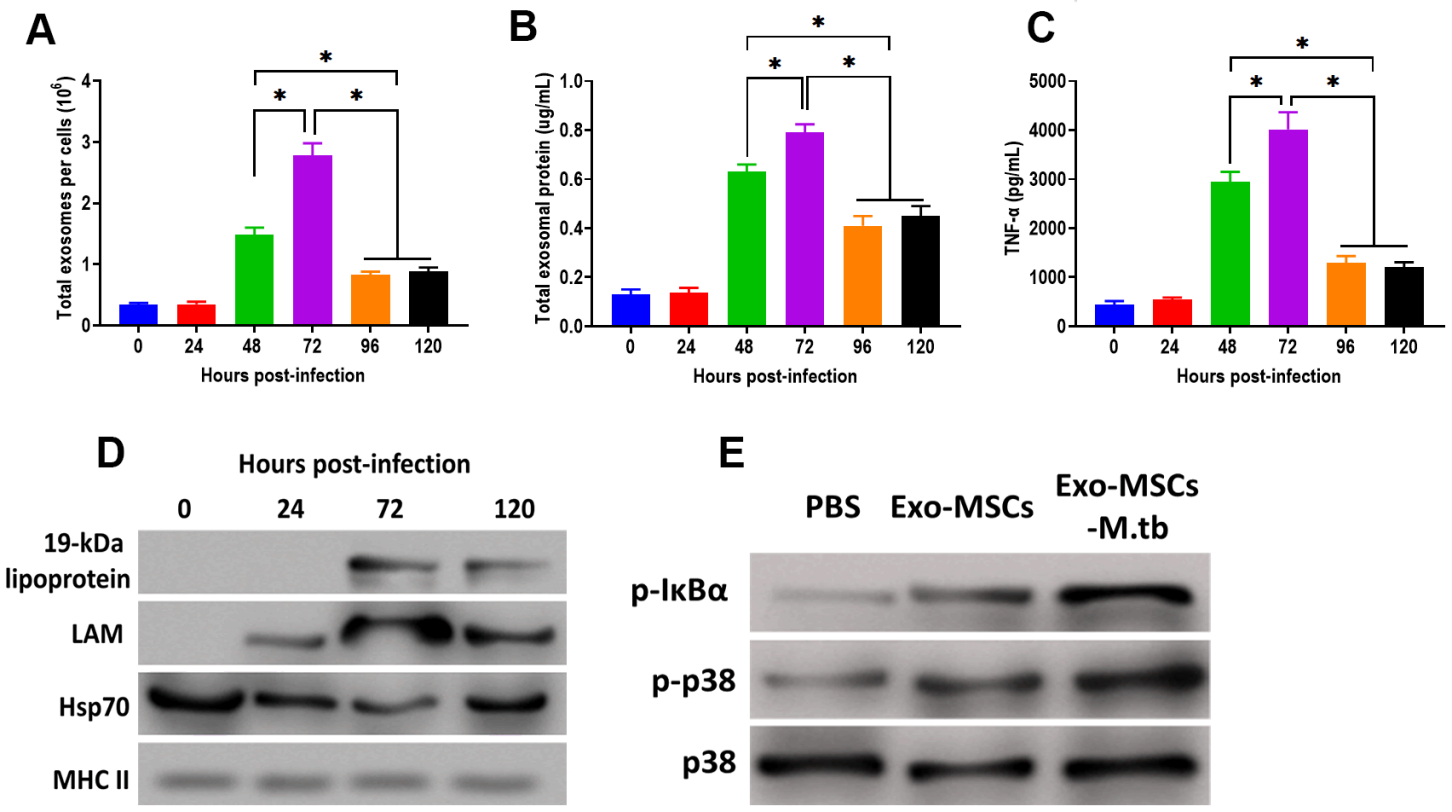
**Pro-inflammatory response induced by Exo-MSCs-M.tb were time-dependent in macrophages.** (**A**) The number of exosomes released from M.tb-infected MSCs at different time points post-injection. (**B**) Exosomal protein in M.tb-infected MSCs. at different time points post-injection. (**C**) TNF-α levels in macrophages treated with Exo-MSCs-M.tb (20μg) at different time points post-injection, as detected by ELISA assay 24 hours after exosome treatment. (**D**) The protein levels of 19-kDa lipoprotein, LAM, Hsp70, and MHC II in exosomes isolated from M.tb-infected MSCs at different time points post-injection, (**E**) The levels of phosphorylated IκBα or p38, and total p38 in macrophages treated with exosomes derived from MSCs or M.tb-infected MSCs, as detected by western blotting assay. *p < 0.05.

After M.tb infection, the ability of exosome releasing of MSCs displayed a time-dependent matter. We found that the number of exosomes and exosomal protein concentration were significantly increased at 48 hours post-infection and reached the highest level after 72 hours of M.tb infection (Figure 5A, 5B). In addition, the effect of Exo-MSCs-M.tb on the pro-inflammatory response also replied on when Exo-MSCs-M.tb was isolated after infection. The results revealed that Exo-MSCs-M.tb isolated at 72 hours post-infection induced the highest level of the pro-inflammatory response (Figure 5C). Meanwhile, Exo-MSCs-M.tb obtained at 24, 96, and 120 hours post-infection failed to initiate the increase of TNF-α of macrophages. Furthermore, the highest levels of LAM and 19-kDa lipoprotein of Exo-MSCs-M.tb were found at 72 hours post-infection (Figure 5D), suggesting that the mycobacterial components were trafficked to Exo-MSCs-M.tb after M.tb infection. Moreover, we observed that the phosphorylation levels of IκBα and p38 were increased in macrophages treated with Exo-MSCs-M.tb, compared with PBS and Exo-MSCs (Figure 5E), suggesting that MAPK and NF-κB pathways are involved in the pro-inflammatory response induced by Exo-MSCs-M.tb.

### Exo-MSCs-M.tb induced pro-inflammatory response through TLRs

**Figure 6:**
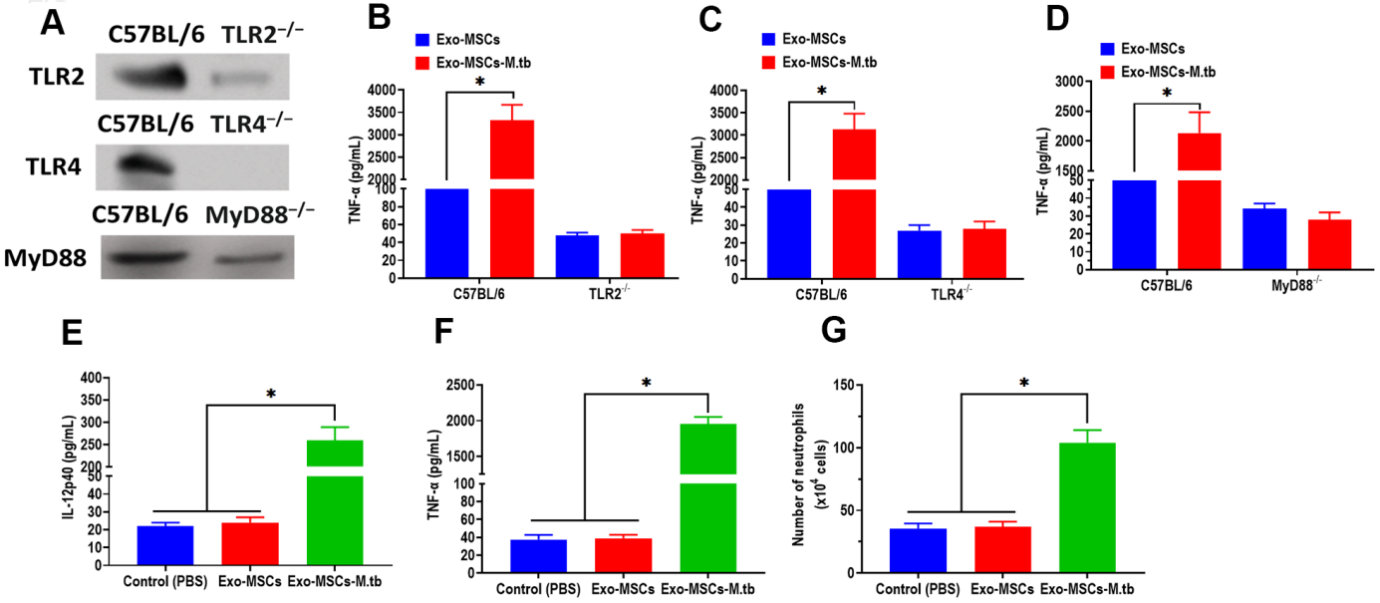
**Exo-MSCs-M.tb induced pro-inflammatory response through TLRs.** (**A**) The protein levels of TLR2, TLR4, and MyD88 in the lung of TLR2^-/-^, TLR4^-/-^, and MyD88 ^-/-^ mice, respectively. (**B**) TNF-α levels in the lung of TLR2^-/-^ mice treated with Exo-MSCs-M.tb (20μg) (20μg), as detected by ELISA assay 24 hours after exosome treatment. (**C**) TNF-α levels in the lung of TLR4^-/-^ mice treated with Exo-MSCs-M.tb (20μg), as detected by ELISA assay 24 hours after exosome treatment. (**D**) TNF-α levels in the lung of MyD88 ^-/-^ mice treated with control (PBS), Exo-MSCs (20μg), and Exo-MSCs-M.tb (20μg), as detected by ELISA assay 24 hours after exosome treatment. (**E**) IL-12 p40 levels in the lung of C57BL/6 mice treated control (PBS), Exo-MSCs (20μg), and Exo-MSCs-M.tb (20μg). (**F**) TNF-α levels in the lung of C57BL/6 mice treated with control (PBS), Exo-MSCs (20μg), and Exo-MSCs-M.tb (20μg). (**G**) Neutrophil infiltration of C57BL/6 mice treated with control (PBS), Exo-MSCs (20μg), and Exo-MSCs-M.tb (20μg). *p < 0.05.

Given the function of TLRs in mediating both MAPK and NF-κB pathways, we then attempted to investigate the effect of TLRs in the pro-inflammatory response of macrophages. We collected macrophages from TLR2^-/-^, TLR4^-/-^, and MyD88^-/-^ mice, respectively. The western blotting assay revealed that the protein expressions of TLR2, TLR4, and MyD88 were significantly lower than those of control mice (Figure 6A). Intriguingly, Exo-MSCs-M.tb failed to induce the pro-inflammatory response in macrophages isolated from TLR2^-/-^, TLR4^-/-^, and MyD88^-/-^ mice, respectively (Figure 6B–6D), which indicates the importance of TLRs in the pro-inflammatory response of macrophages.

### Exo-MSCs-M.tb induced the pro-inflammatory response *in vivo*

**Figure 7:**
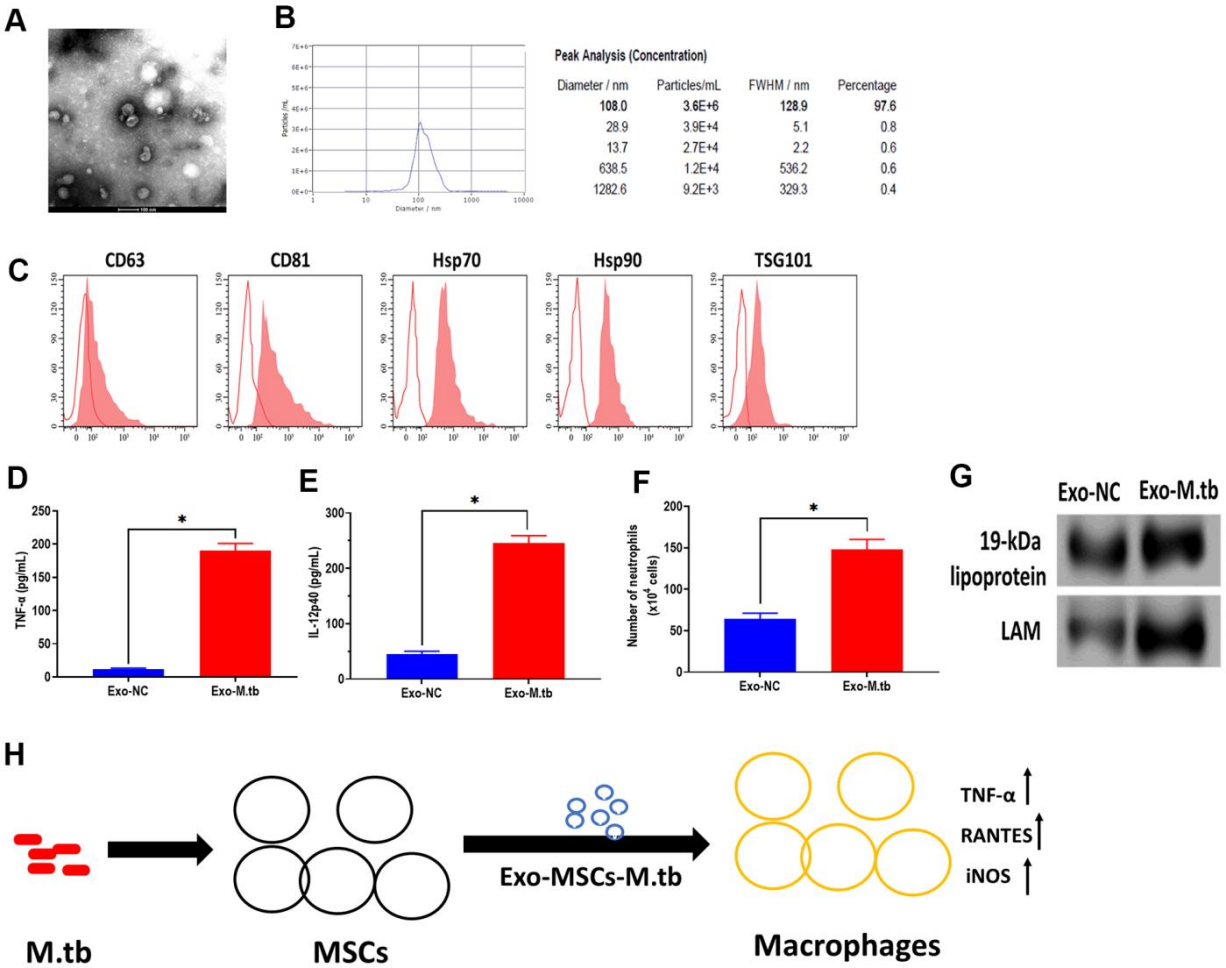
**Exosomes isolated from C57BL/6 mice treated with Exo-MSCs-M.tb induced pro-inflammatory response *in vitro*.** (**A**) Morphology of exosomes isolated from C57BL/6 mice treated with Exo-MSCs-M.tb, as imaged by TEM. Scale bars: 100 μm. (**B**) Concentration and diameter distribution of exosomes isolated from C57BL/6 mice treated with Exo-MSCs-M.tb, as detected by NTA. (**C**) Detection of exosomal markers CD63, CD81, Hsp70, Hsp90, and TSG101, as detected by flow cytometer. (**D**) TNF-α levels in the lung of C57BL/6 mice injected with BALF-derived exosomes (20μg) isolated from mice treated with Exo-MSCs-M.tb (Exo-M.tb) or mice treated with negative control PBS (Exo-NC). (**E**) IL-12p40 levels in the lung of C57BL/6 mice injected with BALF-derived exosomes (20μg) isolated from mice treated with Exo-MSCs-M.tb (Exo-M.tb) or mice treated with negative control PBS (Exo-NC). (**F**) Neutrophil infiltration of C57BL/6 mice injected with BALF-derived exosomes (20μg) isolated from mice treated with Exo-MSCs-M.tb (Exo-M.tb) or mice treated with negative control PBS (Exo-NC). (**G**) The protein levels of 19-kDa lipoprotein, LAM, Hsp70 in BALF-derived exosomes (20μg) isolated from mice treated with Exo-MSCs-M.tb (Exo-M.tb) or mice treated with negative control PBS (Exo-NC). (**H**) Graphic summary. *p < 0.05.

To further determine whether Exo-MSCs-M.tb can induce the pro-inflammatory response *in vivo*, C57BL/6 mice were injected intranasally with PBS, Exo-MSCs, and Exo-MSCs-M.tb. The levels of cytokine IL-12p40 and TNF-α in the lungs were investigated at 0 and 3 days post-injection. The results showed that both IL-12p40 and TNF-α were significantly increased in mice treated with Exo-MSCs-M.tb, compared with PBS and Exo-MSCs (Figure 6E, 6F). More neutrophils were also observed in Exo-MSCs-M.tb-treated mice, relative to those injected with PBS and Exo-MSCs (Figure 6G). Meanwhile, we collected exosomes from the bronchoalveolar lavage fluid (BALF) of C57BL/6 mice treated with Exo-MSCs-M.tb at 5 days post-injection. Through TEM and NTA assays, vesicles isolated from BALF displayed a round shape with a diameter of 108.0 nm (97.6%) (Figure 7A, 7B). Also, exosomal surface marker CD63, CD81, Hsp70, Hsp90, and TSG101 were positively expressed in the vesicles (Figure 7C). These results together confirmed the identification of exosomes. Then, we injected BALF-derived exosomes into C57BL/6 mice, and we found the level of IL-12p40, TNF-α, and neutrophil infiltration were increased in the lungs, suggesting BALF-derived exosomes can also induce the pro-inflammatory response *in vivo* (Figure 7D, 7E). Furthermore, the protein levels of 19-kDa lipoprotein and LAM of BALF-derived exosomes were higher than those of control exosomes, suggesting that the mycobacterial components were trafficked in BALF-derived exosomes (Figure 7G).

## DISCUSSION

MSCs possess regenerative and self-renewal properties and release chemokines, cytokines, and growth factors to maintain homeostasis, repair injured tissues, and suppress inflammation [36]. In general, MSCs act as suppressors for immune activities of T cells, dendritic cells, and macrophages, providing a protective microenvironment for drug-resistant M.tb [37]. Since MSCs are accessible for growing *in vitro* conditions and can be genetically or pharmacologically modified [38, 39], MSCs-based immunotherapeutic strategies are promising for developing the treatment of TB. Accumulating evidence demonstrates that MSCs-derived exosomes have significant therapeutic potential for various diseases, such as liver fibrosis [40], Parkinson's disease [41], traumatic brain injury [16], and cancers [42]. To the best of our knowledge, few studies report the effect of exosomes derived from MSCs on macrophages. In this study, we found that M.tb infection promoted the production of exosomes but had no effect on the MSCs proliferation. Exo-MSCs-M.tb were taken up by macrophages and induced the pro-inflammatory response of macrophages through increasing production of TNF-α, RANTES, and iNOS. Also, we demonstrated that the effect of Exo-MSCs-M.tb was mediated through TLR2/4 and MyD88. Moreover, Exo-MSCs-M.tb could induce the pro-inflammatory response in mice *in vivo*, and exosomes isolated from BALF of Exo-MSCs-M.tb-treated mice could also activate the inflammatory response (Figure 7H).

The release of exosomes is associated with a series of complex processes, including multivesicular bodies (MVBs) transportation, docking, and fusion with plasma membranes [43, 44]. Due to different origins, bioactive components wrapped in exosomes, such as protein or microRNAs, vary between different cell types [45]. For antigen-presenting cells, exosomes derived from dendritic cells or B lymphocytes enrich with costimulatory molecules CD80, adhesion molecule CD54, as well as major histocompatibility complex (MHC) class I and II molecules [46]. In this stud, we found that Exo-MSCs-M.tb positively expressed MHC II and Hsp70 and enriched microbial components lipoarabinomannan (LAM) and 19-kDa lipoprotein. These results suggest that the mycobacterial components were wrapped to Exo-MSCs-M.tb derived from M.tb-infected MSCs.

Growing evidence have been demonstrated that exosomes play a dual role in immune responses, dampening or activating the immune system [47, 48]. In cancers, exosome-mediated signals modulate the immune system by suppressing the actions of T cells and normal killer cells and restraining the differentiation of precursors toward mature antigen-presenting cells [47]. Meanwhile, mature dendritic cell-derived exosomes enriched MHC class II, B7.2, lysosomal-associated membrane protein 1 (LAMP-1) can significantly activate T cells, compared with those derived from immature dendritic cells [49]. Since the function of exosomes displays case-dependent property, exploring the mechanism underlying the function of exosomes should focus on the molecules encompassed in exosomes in future studies.

Given the observation that Exo-MSCs-M.tb enriched with LAM and 19-kDa lipoprotein, pathogen-associated molecular patterns (PAMPs) [50], we speculated that the effect of Exo-MSCs-M.tb might be associated with TLRs signaling pathway that is associated with the role of PAMPs in the immune system [51]. In the present study, we isolated macrophages from TLR2-, TLR4- and MyD88-deficient mice and found that Exo-MSCs-M.tb failed to induce the pro-inflammatory response of macrophages, suggesting that these signals are essential for mediating the effect of Exo-MSCs-M.tb on macrophages. TLRs are type I transmembrane proteins and function in the innate immune system through recognizing microbial structures [51]. Of which, both TLR2 and TLR4 are involved in lipopolysaccharide (LPS)-mediated signaling [51]. Also, MyD88 has been demonstrated to be a common adaptor protein for TLRs signaling [52]. Collectively, TLR2/4 and MyD88 synergistically mediate the signaling of Exo-MSCs-M.tb, eventually inducing a pro-inflammatory response. For future direction, the mechanism in which how these factors work and what exosomal components activate these pathways should be addressed.

In conclusion, we first demonstrated that Exo-MSCs-M.tb induced the pro-inflammatory response of macrophages *in vitro* and *in vivo* and that TLRs signaling is involved in the effect of Exo-MSCs-M.tb on macrophages. This study provides new insight into the potential of MSCs-derived exosomes for the treatment of TB.
